# Evaluation of Lethal Giant Larvae as a Schistosomiasis Vaccine Candidate

**DOI:** 10.1155/2016/4680812

**Published:** 2016-11-09

**Authors:** Yufan Cao, Hongbin Qiao, Yanli Shi, Yu Han, Jinming Liu, Hao Li, Ke Lu, Jiaojiao Lin, Yamei Jin

**Affiliations:** ^1^Shanghai Veterinary Research Institute, Chinese Academy of Agricultural Sciences, Key Laboratory of Animal Parasitology, Ministry of Agriculture, Shanghai 200241, China; ^2^Jiangsu Coinnovation Center for Prevention and Control of Important Animal Infectious Diseases and Zoonoses, Yangzhou, Jiangsu Province 225009, China

## Abstract

Schistosomiasis is a neglected tropical disease of humans, and it is considered to be the second most devastating parasitic disease after malaria. Eggs produced by normally developed female worms are important in the transmission of the parasite, and they responsible for the pathogenesis of schistosomiasis. The tumor suppressor gene* lethal giant larvae (lgl)* has an essential function in establishing apical-basal cell polarity, cell proliferation, differentiation, and tissue organization. In our earlier study, downregulation of the* lgl* gene induced a significant reduction in the egg hatching rate of* Schistosoma japonicum (Sj)* eggs. In this study, the* Sjlgl* gene was used as a vaccine candidate against schistosomiasis, and vaccination achieved and maintained a stable reduction of the egg hatching rate, which is consistent with previous studies, in addition to reducing the worm burden and liver egg burden in some trials.

## 1. Introduction

Schistosomiasis (an infection caused by the blood fluke schistosomes) is the second most common, after malaria, parasitic disease of human and animals worldwide, and it occurs mainly in developing countries. Despite decades of control, there are still millions of people at risk of contracting this infection [1]. Most current schistosomiasis control strategies are based on the use of safe and effective drugs, such as praziquantel and oxamniquine, but these do not prevent reinfection, and the number of infected people has remained constant [2]. The best long-term strategy for the control of schistosomiasis is through immunization with a schistosomiasis vaccine in combination with drug treatment [3]. To date, candidate antigens suggested by the World Health Organization and reported by researchers are not sufficiently protective to be used in the clinic [4]; therefore, it is necessary to search for new, highly protective vaccine candidates.

Lethal giant larvae (LGL) is a member of the SCRIB complex, which interact to regulate the polarity of cells. LGL is a critical molecular integrator of apical and basolateral activities. The WD40 repeats of LGL form *β*-propellers that can act as protein-interacting modules for SCRIB, and the atypical protein kinase C- (aPKC-) mediated phosphorylation of LGL during epithelial polarity establishment is needed for its basolateral localization [5–9]. Many studies have shown that LGL function is essential for the development of polarized epithelia [7, 10–12], localization of cell-fate determinants, and association with the cytoskeletal complex [13, 14]. LGL was first described in the fruit fly* Drosophila melanogaster* as a neoplastic tumor suppressor gene [15, 16]. The tumorous phenotype of the fruit fly is a giant larva [8].

Evolutionarily conserved homologs of* lgl* have been identified in many species, including human, mouse, and worm [17–19] and, in our earlier study, we found that downregulation of the* Sjlgl *gene affects not only egg hatching but also parasite morphology and that SjLGL mostly localizes to the tegument of adult* S. japonicum*, even though SjLGL does not contain a signal peptide or transmembrane domain [20]. In this study, we evaluated SjLGL as a candidate schistosomiasis vaccine. The results showed that specific antibodies were induced following immunization with recombinant SjLGL (rSjLGL), and there were reductions of the egg hatching rate, worm burden, and liver egg burden, although these were not observed in every trial.

## 2. Materials and Methods

### 2.1. Molecular Characterization of* Sjlgl*

The 5333 bp* Sjlgl* sequence was submitted to GenBank (accession number KF246684) and the selected amino acid sequence in this study was translated from bp 1866–2711. The sequence was analyzed as follows. Signal peptide prediction was performed with the SignalP 3.0 server (http://www.cbs.dtu.dk/services/SignalP/). Transmembrane helices were analyzed using the TMHMM server version 2.0 (http://www.cbs.dtu.dk/services/TMHMM-2.0/). The molecular weight (MW) and isoelectric point (pI) were calculated using the ExPASy compute tool (http://www.expasy.ch/tools/pitool.html). The amino acid sequences of the LGL protein were obtained from GenBank and aligned using ClustalX software (http://www.clustal.org/).

### 2.2. *Sjlgl* Cloning and Protein Expression

Upstream and downstream oligonucleotides, 5′-CCGGAATTCATAGTCGCTCTAGGCCATTC-3′ and 5′-CCCAAGCTTTCAGTTAAACTTCTTTCGGTG-3′ (EcoRI and HindIII sites are underlined, resp.), were used to amplify the partial* Sjlgl *coding sequence (GenBank accession number AY812588) from bp 1866–2711. The 846 bp fragment was generated by polymerase chain reaction (PCR) and cloned into the pET-28a (+) vector (Novagen, Darmstadt, Germany), and its identity was confirmed by sequencing.

Overexpression of rSjLGL, with an aminoterminal histidine tag, in* Escherichia coli* BL21 (DE3) cells (Tiangen Biotech Co., Ltd., Beijing, China) was induced by treatment with 1 mM isopropyl-*β*-d-thiogalactopyranoside (IPTG) at 37°C for 5 h. Bacteria were harvested by centrifugation at 10,000 ×g for 15 min. The supernatant was discarded, and the pellet was suspended in 50 mL of phosphate-buffered saline (PBS, pH 7.4) and lysed using ultrasound to release the fusion protein. Following centrifugation at 10,000 ×g for 15 min, the inclusion body protein was extracted in PBS containing urea and purified by passage through Ni-NTA His-Bind Resin (Qiagen GmbH, Hilden, Germany).

### 2.3. Immunization of Mice

Six-week-old male BALB/c mice were divided into two groups of 10 mice each. Animals in the experimental group were injected subcutaneously with 50 *µ*g of rSjLGL fusion protein on d 0, 15, and 30. The recombinant protein was formulated with Montanide ISA 206 (Guoyao, Shanghai, China) at a ratio of 46 : 54. Animals in the control group were administered adjuvant in PBS using the same immunization protocol. The experiments were repeated five times.

Animal care and experimental procedures were conducted in accordance with the guidelines of the Shanghai Veterinary Research Institute for the Care and Use of Laboratory Animals.

### 2.4. Challenge Infection and Worm Burden Recovery

Two weeks after the last boost, mice were infected via exposure of abdominal skin for at least 15 min to water containing 40 ± 5 cercariae. At 42 d after infection, mice were sacrificed to recover parasites by perfusion via the hepatic vein, and their livers were collected. The worm burden reduction was calculated as [21]

(1) $$\begin{array}{c} PL=\left(\;1-\frac{WREG}{WRCG}\;\right)\times 100\%, \end{array}$$

where PL is the protection level; WRCG is the number of worms recovered from the control group; and WREG is the number of worms recovered from the experimental group.

### 2.5. Liver Egg Count and Miracidium Hatching Rate

To evaluate the liver egg burden, the liver was weighed and homogenized, and 1 mL of homogenate was mixed with 1 mL of 10% (w/v) NaOH and heated for 10–15 min at 56°C. The eggs were counted, and the number of eggs/g was compared to the control mice, using the following formula:

(2) $$\begin{array}{c} PL=\left(\;1-\frac{EPGEG}{EPGCG}\;\right)\times 100\%, \end{array}$$

where EPGCG is the number of eggs/g of the control group and EPGEG is the number of eggs/g of the experimental group.

To hatch the miracidia, 4 mL of liver homogenate was added to a flask filled with chlorine-free water. The neck of the flask was filled with a very thin layer of absorbent cotton (avoiding air bubbles, which could obstruct the miracidia) and kept at 27°C in the light. The supernatant above the cotton, which included the miracidia, was collected at 2 h and at 4 h after hatching. The miracidia were fixed with iodine and collected by centrifuging the supernatant at 4000 ×g for 5 min at 4°C and counted under a light microscope (magnification 40x). The average egg hatching rate was calculated as miracidium/added egg, and the reduction in the hatching rate was calculated by comparing the results to the control group:

(3) $$\begin{array}{c} PL=\left(\;1-\frac{HREG}{HRCG}\;\right)\times 100\%, \end{array}$$

where HRCG is the egg hatching rate of the control group and HREG is the egg hatching rate of the experimental group.

### 2.6. Measurement of Anti-SjLGL Antibodies

Following immunization, sera were collected at 0, 15, 30, and 45 d after the first immunization (2 weeks after each immunization) for measurement of IgG, and sera were collected at 0, 10, 25, and 40 d after the first immunization (10 d after each immunization) for measurement of IgE. Specific anti-SjLGL antibodies were measured using an indirect enzyme-linked immunosorbent assay (ELISA). First, 96-well microtiter plates were coated with 100 *µ*L of 10 *µ*g/mL rSjLGL overnight at 4°C and then blocked for 2 h at room temperature with 150 *µ*L/well of PBS containing 1.5% (w/v) bovine serum albumin. A 100 *µ*L volume of each serum sample was diluted 1 : 100 (v/v) in PBS, added to each well, and incubated for 1 h at room temperature. Plate-bound antibody was detected using peroxidase-conjugated anti-mouse IgG (Dingguo, Shanghai, China) or anti-mouse IgE (AbD Serotec, Raleigh, NC, USA) diluted in PBS 1 : 5,000 (v/v) or 1 : 1,000 (v/v), respectively.

### 2.7. Statistical Analysis

Data are expressed as mean ± standard deviation (SD). Statistical analysis was done with Student's* t*-test or one-way analysis of variance. The level of a statistically significant difference was set at *p* < 0.05.

## 3. Results

### 3.1. Sequence Analysis of* Sjlgl*

The posttranslational modifications of the complete 5333 bp* Sjlgl* sequence were predicted. The results showed that* Sjlgl* encodes a protein of 1435 amino acid residues, with a predicted molecular mass of ~155.68 kDa and a pI of 5.02; the protein does not contain a signal peptide or transmembrane helix.

**Figure 1:**
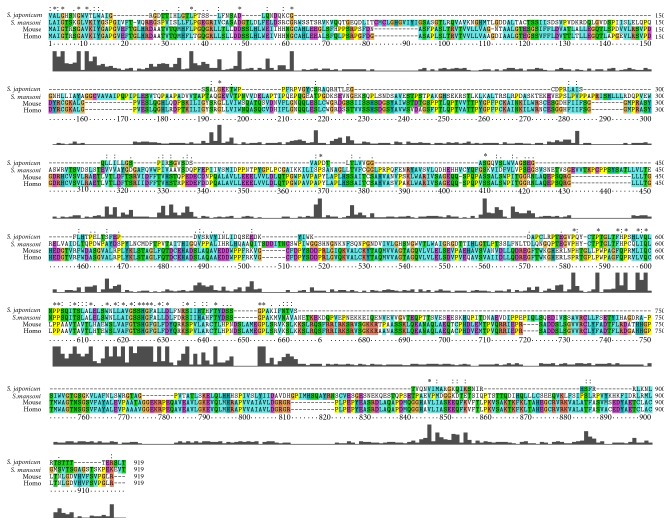
The selected protein sequence of SjLGL in relation to* S. mansoni*,* M*.* musculus*, and* H. sapiens* LGL sequences. ClustalX alignment of the derived amino acid sequences of SmLGL (XP_002577079.1), MmLGL (NP_001152876), and HsLGL (NP_004131.3). Regions with a high level of identity and similarity between the LGL sequences are shown in color.

The 849 bp sequence of the selected* Sjlgl* segment encodes a protein of 282 amino acid residues, with a predicted molecular mass of ~30.76 kDa and a pI of 6.85. A comparison of the amino acid sequences showed that the LGL segment of* S. japonicum *that was selected in our experiment shared 86%, 36%, and 33% identity with its orthologs in* Schistosoma mansoni*,* Mus musculus*, and* Homo sapiens*, respectively (Figure 1).

### 3.2. Production of rSjLGL

**Figure 2:**
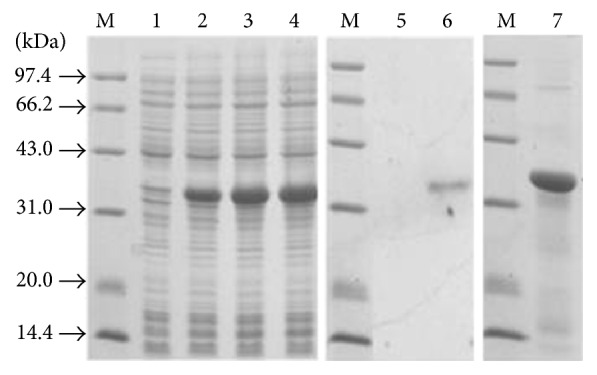
Expression and purification of rSjLGL in* E. coli*. Cell extracts and fractions from* E. coli* BL21 (DE3) cells that were transformed with the pET28-Sjlgl vector were separated by 12% SDS-PAGE. Lane M: size markers. Lane 1: total extract of a clone before induction. Lanes 2, 3, and 4: total extracts of a clone 2, 4, and 6 h after induction with 1 mM IPTG. Lanes 5 and 6: western blot using a polyclonal anti-rSjLGL antibody (lane 5: antigen (total extract of a clone before IPTG induction); lane 6: total extract of a clone after 5 h of IPTG induction; lane 7: protein purified from the supernatant by passage through Ni-NTA His-Bind Resin).

The partial sequence from bp 1866–2711 of* Sjlgl* was obtained by PCR amplification, cloned into the pET28 expression vector, and expressed in* E. coli* BL21 (DE3) cells via IPTG induction. During sodium dodecyl sulfate-polyacrylamide gel electrophoresis (SDS-PAGE) (12% (w/v) polyacrylamide), the histidine-tagged protein ran as a single band with a MW of ~36 kDa (Figure 2), which is in accordance with the predicted MW of the rSjLGL protein. Bacteria were lysed using ultrasound, and the lysate was separated into soluble and insoluble fractions; the insoluble fraction contained the majority of the recombinant protein. Next, the soluble protein was purified by passage through Ni-NTA His-Bind resin, and the main recombinant protein in the eluted fractions was pooled (Figure 2, lane 7). After purification, the concentration of protein was ~5 mg/mL, and this was used to immunize mice.

### 3.3. Evaluation of Anti-SjLGL IgG and IgE Antibodies

**Figure 3:**
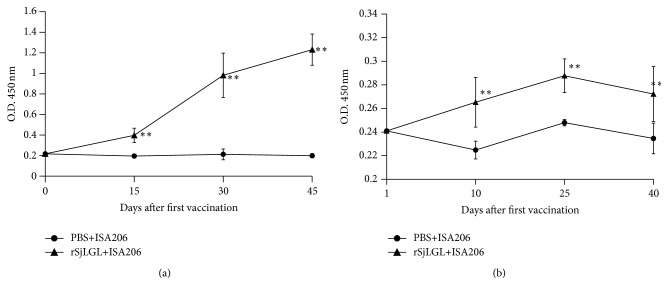
Kinetics of the induction of specific anti-rSjLGL IgG and IgE antibodies in immunized mice. (a) Sera of 10 immunized mice/group were collected on d 0, 15, 30, and 45 after the first immunization and analyzed by ELISA. (b) Sera of 10 immunized mice/group were collected on d 0, 10, 15, and 40 after the first immunization and analyzed by ELISA. The results are presented as the mean absorbance at 450 nm (*A*_450_) of one trial measured for one experiment for each group. Statistically significant differences in the antibody levels of vaccinated mice compared with those of the PBS + adjuvant control group are denoted by ^*∗∗*^(*p* < 0.01).

To evaluate the presence of anti-SjLGL IgG and IgE antibodies, sera from 10 vaccinated mice from each group were tested using an indirect ELISA. Significant titers of specific anti-SjLGL IgG antibodies were detected at all time points tested after the first immunization. The results of the protein immunization experiment showed that the IgG level increased to a high level after the second immunization and remained at that level (Figure 3(a)). The IgE level increased slowly after the first immunization, and it was significantly higher than that of the PBS control group (Figure 3(b)).

### 3.4. SjLGL Immunization Induces a Protective Effect

To measure the protection levels induced by SjLGL immunization, mice were challenged with 40 ± 5 cercariae, and reductions of the worm burden, liver egg burden, and egg hatching rate were determined (Table 1).

Protein vaccination was repeated five times, and, following immunization, worm burden and liver egg burden reductions were observed in two and three, respectively, of the five experimental tests; while the worm burden and liver egg burden reductions did not significantly differ from those of the control group in the other tests, the egg hatching rate reductions were quite similar in these tests, and they reached 75.84% (Table 1).

## 4. Discussion

Schistosomiasis continues to be a significant global public health concern, and an effective control strategy will require the development of effective vaccines. In recent reports, the use of recombinant antigens, for example, Sm-TSP-2 and Sm29 induced ~50% reductions of the worm burden, thereby demonstrating that a vaccine against schistosomes is achievable [21, 22], which led us to search for additional vaccine candidates.

The* lethal giant larvae (lgl)* gene is well conserved among species, and it functions to link apical-basal cell polarity regulation to cell proliferation control in epithelial tissues [11, 23]. Mutation of* lgl* can cause cellular overproliferation, leading to tissue overgrowth (tumors). Mutation of* lgl* in* D. melanogaster* results in the loss of apical-basal polarity and uncontrolled proliferation, which results in neoplastic tumors [11, 24, 25]. The* lgl* gene has a remarkable impact on the larval stage in flies and it makes large maternal contributions. Homozygous* lgl* mutant embryos from a heterozygous mother will progress into the larval stage and then die because of polarity defects when the* lgl* products from the mother are exhausted. Based on our RNA interference experiments, we think LGL plays important roles in the development of* S*.* japonicum* embryos [20]. Additionally, we found that the partial sequence from 1866–2711 bp of* Sjlgl* is conserved in mammalian* lgl* genes; thus, its potential as a vaccine candidate was investigated. In this study, immunization with SjLGL achieved a stable and significant reduction of the egg hatching rate; the reduction of the egg hatching rate was 75.84%, and it remained stable at >50%. The effect of SjLGL immunization on the egg hatching rate might be related to the function of LGL in cell polarity regulation. We did not observe a statistically significant reduction of the worm burden or the liver egg burden in each trial. We believe that differences among individual mice were related to differences in the number and the male/female ratio of cercariae, although the reduction of the egg hatching rate was statistically significant and stable. We tested the combined DNA plus protein vaccination strategy in this study because a similar strategy yielded good results in an HIV vaccine study [26]; however, the combined vaccination strategy did not exhibit greater protective efficacy than a single-protein vaccination strategy (data not shown). This may be because viruses and parasites induce different immune responses.

A mature female* S. japonicum* discharges 500–3500 eggs/d [27], which is important for pathological changes in the host liver and, together with the egg hatching rate, is responsible for spreading schistosomiasis. Immature eggs that are newly deposited in the host liver by female worms do not result in granuloma formation, and they do not induce serious pathological changes. The eggs mature after 10-11 d of development; the miracidium in mature eggs will secrete some soluble egg antigen, which causes the granulomatous lesions and hepatomegaly. In the present study, immunization with SjLGL resulted in a statistically significant and stable reduction of the egg hatching rate. In our earlier study, vaccinating mice with SjLGL generated a Th1-type immune response, which protects against schistosomiasis [28]. Additionally, in this study, not only did vaccination with SjLGL induce a statistically significant increase in the IgG antibody titer, but also vaccination with SjLGL significantly increased the IgE antibody titer, which is important because the IgE response to adult worm antigens is related to resistance to reinfection [29, 30]. Together, these results suggest that SjLGL is a candidate vaccine to control schistosomiasis by reducing the liver egg burden and the egg hatching rate.

## Figures and Tables

**Table 1:** Protection level induced in BALB/c mice following immunization with rSjLGL and infection with 40 cercariae.

	Adult worms mean ± SD (% reduction)	Liver eggs mean ± SD (% reduction)	Egg hatching rate (% reduction)
Test 1			
PBS+ISA206	12 ± 5.78	15446.27 ± 4518.95	6.43 ± 2.44
rSjLGL+ISA206	12.6 ± 6.22	17286.02 ± 5933.09	1.55 ± 0.79
	(−7.57)	(−11.91)	(75.84^*∗∗*^)

Test 2			
PBS+ISA206	14.5 ± 5.19	60374.84 ± 26931.24	2.42 ± 0.96
rSjLGL+ISA206	18.2 ± 2.94	64699.45 ± 32697.79	1.09 ± 0.29
	(−25.51)	(−7.16)	(54.62^*∗∗*^)

Test 3			
PBS+ISA206	13.9 ± 5.68	35537.82 ± 17296.66	4.20 ± 2.13
rSjLGL+ISA206	12.66 ± 5.14	17820.01 ± 8997.366	1.96 ± 0.90
	(8.87)	(49.86^*∗∗*^)	(53.24^*∗∗*^)

Test 4			
PBS+ISA206	22 ± 8.07	30513.28 ± 13690.01	4.15 ± 2.6
rSjLGL+ISA206	11 ± 2.7	25081.94 ± 15991.31	1.59 ± 1.01
	(50.0^*∗*^)	(33.47^*∗*^)	(61.71^*∗*^)

Test 5			
PBS+ISA206	17.14 ± 3.76	50135.58 ± 7204.55	7.57 ± 3.43
rSjLGL+ISA206	14.92 ± 6.19	35478.2 ± 9444.48	3.44 ± 1.19
	(12.91^*∗*^)	(29.23^*∗*^)	(54.49^*∗*^)

Tests 1–5, *n* = 10. ^*∗*^Statistically significant compared with the control group (*p* < 0.05), ^*∗∗*^(*p* < 0.01).
